# Group plus “mini” individual pre-test genetic counselling sessions for hereditary cancer shorten provider time and improve patient satisfaction

**DOI:** 10.1186/s13053-020-0136-2

**Published:** 2020-02-19

**Authors:** Jaclyn Hynes, Andrée MacMillan, Sara Fernandez, Karen Jacob, Shannon Carter, Sarah Predham, Holly Etchegary, Lesa Dawson

**Affiliations:** 1 Clinical Epidemiology, Faculty of Medicine, Memorial University, St. John’s, Newfoundland and Labrador Canada; 2 Provincial Medical Genetics Program, Health Sciences Centre, Eastern Health Authority, St. John’s, Newfoundland and Labrador Canada; 3 Gynecologic Oncology, Faculty of Medicine, Memorial University, St. John’s, Newfoundland and Labrador Canada

**Keywords:** Genetic counseling, Inherited cancers, Hereditary breast ovarian, Lynch syndrome

## Abstract

**Background:**

Genetic counselling (GC) is an integral component in the care of individuals at risk for hereditary cancer predisposition syndromes (CPS). In many jurisdictions, access to timely counselling and testing is limited by financial constraints, by the shortage of genetics professionals and by labor-intensive traditional models of individual pre and post-test counselling. There is a need for further research regarding alternate methods of GC service delivery and implementation. This quality improvement project was initiated to determine if pretest group GC followed immediately by a ‘mini’ individual session, would be acceptable to patients at risk for hereditary breast and colon cancer.

**Methods:**

Patients on waitlists for GC at the Provincial Medical Genetics Program in St. John’s, NL, Canada (*n* = 112), were contacted by telephone and offered the option of a group counselling session (GGC), followed by a “mini” individual session, versus (TGC) traditional private appointments. GGC sessions consisted of a cancer genetics information session given to groups of 6–20 followed by brief 20 min “mini” individual sessions with the patient and genetic specialist. TGC individual appointments provided the same cancer genetics information and counselling to one patient at a time in the classic model. All but 2 participants selected group+mini session. A de-identified confidential 12-item, Likert scale survey was distributed at the conclusion of mini-sessions to measure perceptions of GGC and satisfaction with this counselling model.

**Results:**

Sixty participants completed questionnaires. The majority of participants strongly agreed that they were comfortable with the group session (58/60); the explanation of cancer genetics was clear (54/59); they understood their cancer risks (50/60); and they would recommend such a session to others (56/59). 38/53 respondents disagreed or strongly disagreed that they would prefer to wait for a traditional private appointment. All 5 participating genetic counselors reported a preference for this model. At the end of the pilot project, the waitlist for counselling/testing was reduced by 12 months.

**Conclusions:**

Group pre-test genetic counselling combined with immediate “mini” individual session is strongly supported by patients and reduces wait times. Additional formal investigation of this approach in larger numbers of patients is warranted.

## Background

Hereditary Cancer Predisposition Syndromes (CPS) are an important contributor to the burden of cancer in Canada. Most commonly, these cancers are a result of BRCA-associated breast and ovarian cancer syndromes or hereditary gastrointestinal malignancy syndromes such as Lynch Syndrome [1].

There is significant value in the identification of those with CPS, especially BRCA and Lynch Syndrome. A woman with a pathogenic variant (PV) in *BRCA1 or* 2 has lifetime risk of breast cancer and ovarian cancer of 50–70% and 20–40% respectively, rates which are 5–15 times the usual population rates [2–4]. Detection of these high-risk individuals and implementation of breast MRI surveillance and surgical prevention with risk-reducing oophorectomy or risk-reducing mastectomy is highly effective and has been shown to be cost effective for health systems [5–7]. Implementation of regular 1–2 yearly colonoscopy in those with mismatch repair PV causing Lynch syndrome is associated with a 10-17 year improvement in overall survival [8]. Beyond the prevention of subsequent cancers in the individual and the opportunities for prevention in relatives, the identification of those who carry PVs in these genes has now become important in the tailoring of personalized cancer therapeutics such as PARP inhibitors for ovarian and breast cancers and anti-PD-1 monoclonal antibody-based agents for Lynch Syndrome associated colorectal cancer [9–11].

Pre-test genetic counseling (GC) is the standard of care for individuals at risk for hereditary cancer and assists patients as they make informed medical decisions about testing and cancer risk reduction [12, 13]. The demand for genetic counseling has seen a steady increase over the past two decades, specifically in the domain of inherited cancers [14, 15].

The landscape of genetic testing for cancer predisposition has changed significantly over the last 10 years. Since the advent of next generation sequencing (NGS), multigene panels have become the standard of care and have replaced traditional Sanger sequencing. Many more genes are tested; subsequent updated testing is often required and the complexity of results and disclosure to patients has placed increased pressure on genetics services. Increasing public awareness about cancer genetics has led to dramatic increases in referrals that overwhelm genetics programs across Canada and result in wait times that are unacceptable to the public [16, 17]. These new challenges are impacting efficient access to traditional models of genetic counseling services and highlight the need for alternative counselling models.

Several alternatives to traditional genetic counseling have been investigated, including telephone counselling, pre-counseling education sessions, and group genetic counseling (GGC) [18]. A recent scoping review examined four alternative models of genetic counseling (telephone counseling, tele-genetics, GGC and embedding genetic counselling) [19]. All models improved patient access to genetic services and suggested alternative genetic services are a viable option for reaching a higher volume of patients while maintaining similar levels of patient knowledge and satisfaction when compared to traditional one-on-one private genetic counseling.

Given the nearly three-year waitlist for genetic counseling services in the province of Newfoundland and Labrador (NL), Canada, the Provincial Medical Genetics Program ran a pilot project implementing an alternative genetic counselling model as a way to potentially reduce wait times. This paper describes the implementation of a group+mini individual genetic counselling model and presents patient satisfaction data collected during the initiative.

## Methods

### Aims

This quality improvement initiative aimed to evaluate the perceptions and satisfaction of patients undergoing group genetic counseling prior to genetic testing for cancer predisposition.

### Participants

Patients were identified from two groups on waiting lists of the Medical Genetic Department at Eastern Health Authority, St. John’s, NL, Canada. They were either 1) those with a personal history of breast, colon or endometrial cancer who were eligible for genetic testing by local institutional criteria based on pathology/age at diagnosis or 2) those without cancer who had a known family history suggestive of CPS with an un-referred living relative eligible for testing.

### Procedure

Patients were identified sequentially from the respective waitlist and received a telephone call from the Provincial Medical Genetics Program administrative staff to offer the option of a group counseling session. Individuals were told that the visit would consist of a presentation about cancer genetics for a group of 6–20 patients at the Genetics Department, followed by a mini (~ 20 min) individual session. Patients were advised that they could elect to attend a group session or could opt to wait for their private appointment as per the traditional clinic model. All patients were informed that the content of the information they would receive was similar in both models. It was acknowledged that attendance at a group session might allow for an earlier appointment than a standard private session. There was no formal structure in place to demonstrate how much earlier individuals would be seen for the group session compared to a private appointment, but it was anticipated to be a difference of several months.

Patients who selected the GGC model were scheduled for 1 of 13 sessions between November 2016–October 2017. Lead genetic counsellor (AM) examined patients’ medical charts and pedigrees prior to group counseling. Group sizes ranged from 6 to 20 patients. To increase internal validity and session consistency, the same two individuals (authors AM, LD) facilitated every session; however, additional genetic counsellors were included for larger group sessions. Sessions commenced with a standardized presentation and patients were encouraged to ask questions. The intention was to create a semi-formal environment, providing the opportunity patient-initiated discussions.

Following the group education, ~ 20-min individual sessions were conducted with each participant. Each genetics professional was assigned 3–4 participants who were then seen sequentially for the mini individual session. The mini sessions were offered as a way to review each patient’s history confidentially, to answer question privately, to confirm decisions about testing, to assist the process of referral of affected family members and to complete appropriate consent forms and requisitions. Unaffected participants were counselled about why testing a family member would be the most informative for the family and were offered support in the outreach and referral of that individual.

Quality assurance questionnaires were distributed at visit completion and were collected in an anonymized fashion. Patients were aware that filling out the survey was voluntary and that questionnaires contained no identifying data.

### Patient measures

A 12-item, 5-point Likert scale survey was used to examine patient perception of the group counselling + mini-individual session. Questions were chosen to assess if patients found the sessions helpful, if they felt comfortable with the group session, and if they would recommend group sessions to other patients. Questionnaire items inquired about possible patient preference for a traditional private session and satisfaction with wait time between the group session/mini-individual session. The purpose of this quality assurance questionnaire was to evaluate patient satisfaction and comfort with the counselling experience before the implementation of any group counselling model in our jurisdiction. The Likert scale survey included the following responses: 1 = strongly agree, 2 = somewhat agree, 3 = neither agree nor disagree, 4 = somewhat disagree, 5 = strongly disagree.

### Statistical analysis

SPSS Version 20 was used to analyze the data. Descriptive statistics included means (SD) for variables with normal distributions, medians (interquartile range) for variables with skewed distributions, and n (%) for nominal and ordinal variables.

This project was run by the Provincial Medical Genetics Department, Eastern Health Authority, St. John’s, NL, Canada as a quality assurance initiative.

## Results

### Patients

A total of 112 patients were seen between November 2016 and October 2017. A total of 60 de-identified paper questionnaires were collected and analyzed. At completion of the analysis of these questionnaires, given the overwhelming positive response and lack of observed adverse events, data collection was halted after 60 patients.

### Questionnaire responses

**Fig. 1:**
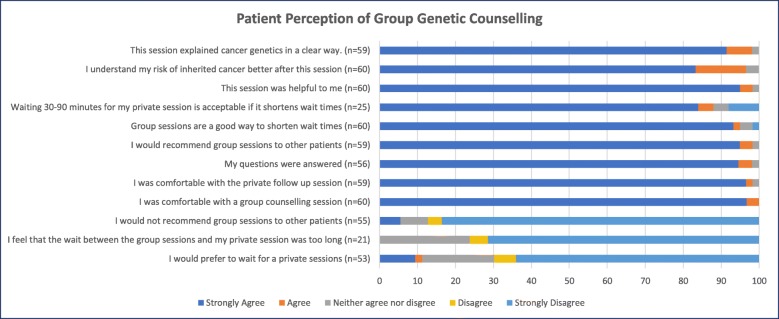
Questionnaire results from patients attending group + mini genetic counseling sessions for hereditary cancer assessment

Responses to all 12 items are reported in Fig. 1. The majority strongly agreed that they were comfortable with the group session (58/60; 97%), that the explanation of cancer genetics was clear (54/59; 92%), that they understood their cancer risks (54/59; 92%), and that the appointment was helpful to them (57/60; 95%). In addition, patients strongly agreed that all questions had been answered (53/56; 95%) and that they would recommend group sessions to other patients (56/59; 95%). All questions received a mean score which correlated to responses which indicated highest support for the group sessions. Questions and descriptive statistics of responses are outlined in Table 1.

**Table 1:** Descriptive statistics

	Mean	Std. Deviation
This session explained cancer genetics in a clear way	1.10	.357
This session was helpful to me	1.07	.312
I understand my risk of inherited cancer better after this session	1.20	.480
I was comfortable with a group counselling session	1.03	.181
I was comfortable with the private follow up session	1.05	.292
My questions were answered	1.07	.322
I would recommend group sessions to other patients	1.07	.314
I would not recommend group sessions to other patients	4.60	1.029
Group sessions are a good way to shorten wait times	1.15	.633
I would prefer to wait for a private session	4.13	1.331
I feel that the wait time between the group session and my private sessions was too long	4.48	.873
Waiting 30–90 min for my private sessions is acceptable if it shortens waitlist times	1.44	1.158

Our team was interested in understanding patients’ experience of the time between their group session and their mini session. Patients were asked “I feel that the wait time between the group session and my private sessions was too long” (4.48 +/− .873) and “Waiting 30-90 minutes for my private session is acceptable if it shortens waitlist times” (1.44 +/− 1.158). Responses indicated patients felt the interval between sessions was acceptable if it shortened wait times for individual appointments.

### Provider time

The total number of provider time hours was not calculated formally during this pilot, however some estimates are possible. Total preparation time was estimated to be 20 h. Each session took 2 hours including the group and “mini” individual appointments for a total of 26 h with 2–4 healthcare providers per session. The initiative required between 90 and 110 h of genetic counselor time and 20 h of physician time. This is contrasted with an anticipated 224 h of provider time required to counsel and test the same number of patients in the traditional model.

## Discussion

This early pilot project provides initial evidence to support the implementation of a new group counselling session model at our center. We sought to ensure that patients were satisfied with their group counselling experience before continuing to implement the program at a larger scale. Initial participants reported comfort and satisfaction with this style of counselling. While formal evaluation data were not collected from the genetic counsellors participating in the initiative, all particpating counsellors reported that they were supportive of the new model and would select it as a preferred method of care delivery in future.

Prior to implementation, our team examined the literature published on the effectiveness and success of GGC for individuals at risk for BRCA mutations and/or Lynch Syndrome. Calzone et al. conducted a pilot, randomized control trial which compared GGC and individual GC methods in those at risk for BRCA1/2 gene mutations [17]. There were no significant differences in self-reported satisfaction between counselling type, and the majority of participants indicated their assigned method provided them with adequate information to make educated genetic testing decisions. Both groups suggested they felt there was sufficient time to ask questions, and participants tended to be equally satisfied with the method they were assigned with a 95% confidence interval for satisfaction scores ranging from 79 to 91% (*p* = 0.82). A comparison between groups showed significantly more time spent per patient in individual sessions (1.25 h) than in GGC sessions (0.74 h; *p* = 0.0001). Correlation between time spent per patient and gain in knowledge was weak (r < 0.30) and the two methods were equivalent with regard to knowledge retention over time. As well, there were no significant differences in knowledge scores between individual and group counselling groups, and the number of individuals who opted not to avail of genetic testing (4%) were equally distributed between groups. The findings from that study are demonstrably similar to the results of this project. Participants indicated they understood their risk of inherited cancer better after the session (1.2 +/− .480) and they were comfortable in the group setting (1.03 +/− .181).

A review published by Buchanan et al. reported that up to 10% of cancer genetic counsellors had used group genetic counselling, and across several studies, GGC decreased time per-patient for genetic counsellors [20]. This review questioned whether cancer patients would widely accept group counselling and ultimately concluded patient satisfaction may be highest when they are allowed to choose the method in which they are counselled. The overwhelming majority of our patients strongly agreed they were happy with the group session (58/60; 97%), and they would recommend group sessions to other patients (56/59; 95%).

Benusiglio et al. included a total of 210 patients for group counselling and were able to demonstrate that this style of counselling session allowed them to see more patients within a four-hour time frame than if patients attended a private session [21]. Decreased provider time has been reported using the group counselling method (1.25 h vs. 0.74 h, *P* = 0.0001) in other studies [17]. Although our project was not designed to calculate reductions in wait time or a provider time per patient, our team reported clearly that this style of counselling allowed them to meet the increased referral demand efficiently. Counsellors observed that the waitlist decreased as a result of the GGC sessions and felt that at the end of the pilot period, patients were being seen faster from the date of referral than before implementation.

Ridge et al. reported high rates of decline for group counselling sessions (40% rejection rate) [22]. This study had a small sample of 42 women, of which 17 declined the invitation for a group counselling session. Patients received one-on-one follow up if requested or determined necessary by the counsellor. Group counselling sessions were not followed with a mini interview at the end of the group session. The high rejection rate may highlight the importance of a mini session.

Rothwell et al. also reported that individuals preferred private session when given the option [18]. Similarly, this study did not include a mini-private session. The genetic counsellors in our initiative decided to add in the private session to ensure that individuals completed their genetic counselling experience with as much information as possible. Benusiglio et al. had a rejection rate of approximately 20% but they did not disclose the option of a private session unless asked specifically by the patient [21]. Our counsellors did not wish to withhold the option of a private session.

Another factor influencing GGC acceptance rates in previous studies may have been the lack of personalized outreach at the time of invitation. Ridge et al. sent study invitations for group sessions by mail [22]. Reasons women listed for rejecting the group counselling session included the need for confidentiality, wanting to bring multiple family members, being a private person, and being intimidated by strangers. Individuals may not have understood or felt comfortable with participation in the GGC in the absence of discussion with staff before participating. This may highlight the importance of personal contact from a team member. This emphasizes that not all individuals will be receptive to this style of counselling, and that it should be clear that individuals may have the option to choose a private session.

The decline rate for our project was substantially lower (approximately < 5% rejection rate), which may be explained by the personalized phone call approach. The RCT by Calzone et al. included a mini interview and still demonstrated a reduction in provider time; therefore, we believe this mix of private and group setting will allow for optimal uptake and satisfaction, along with efficiency [17].

### Study limitations

This project was developed as a local quality improvement initiative to assess patient satisfaction with GGC before developing a plan to implement the model into practice. There are therefore several limitations. This project was not conducted as a prospective REB-approved formal research project; there were no blinding procedures to either the counsellors or patients, nor were there formal research questions or hypotheses being tested. The initiative was launched in response to a dramatic worsening of wait times for counselling in cancer patients that approached 3 years.

The specific identities of those who choose to complete the survey are not known, nor can the pattern of responses be linked to any specific patients’ characteristics such as anxiety about hereditary cancer, educational level, socio-economics status or health literacy. Although a 54% response rate for questionnaire completion is not unexpected for this type of quality assurance project, it is possible that those patients who did not complete the survey had differing opinions about GGC. A future research design addressing this question would measure patient satisfaction outcomes in both traditional and GGC models for comparison.

Patients were given the option to wait for their private appointment, which could have resulted in selection bias. However, almost all patients offered the new model accepted and participated in the group session, so we do not believe selection bias is likely. In addition, the satisfaction survey utilized was not a validated scale and only included 12 questions. Our study was not designed to evaluate the time-effectiveness of GGC in a statistically rigorous manner; this study focused on patient satisfaction with a group method of counselling. All members of the genetic counselling team reported that implementation of GGC sessions reduced the clinic waitlist and allowed them to see more individuals than if all sessions had been private. Despite these limitations, our findings have practical significance for hereditary cancer genetic counsellors. Our counsellors are satisfied with the results of the pilot and intend to implement expanded use of this model.

This approach may be a promising model for health authorities and medical departments as they face the challenge of increased referrals and complex testing. When implementing group sessions in any jurisdiction, personnel must be always mindful of the protection of individual patient privacy and inherent loss of confidentiality that accompanies group participation. Any center considering this strategy will need to explore the local regulatory and privacy legislation landscape. These elements will need to be included in a larger study of the intervention. This project asked patients only if they felt comfortable in the groups and did not include questions specifically about privacy concerns. Individually, no patient expressed concern about confidentiality and in fact, many voiced a preference for the group dynamic in that they were able to hear answers to different questions beyond their own. Patients commented that casual conversations with other participants before and after group sessions provided support and sense of community.

### Practical implications

This quality assurance project provides support for using GGC as a tool to shorten wait times in a public payer system, while continuing to provide high level care. Clinics which may be struggling to manage the increase of referrals may benefit from implementing a similar program at no additional operational cost. This project demonstrates the real life, practical outcomes of implementing group counselling.

## Conclusions

This preliminary evaluation of a group + mini individual genetic counselling model reveals that this approach is highly acceptable to both patients and genetic counselors and is a preferred model of care delivery. Use of group information sessions followed by individual mini sessions allowed for delivery of high-quality genetic counselling and reduced wait times. Larger formalized research projects to evaluate this model in more detail are needed.

## Data Availability

The datasets used and analyzed during the current study are available from the corresponding author on reasonable request.

## Abbreviations

CPS: Cancer Predisposition Syndromes
GC: Genetic Counselling
GGC: Group Genetic Counselling
GGC: Group Genetic Counselling
NGS: Next generation sequencing
NL: Newfoundland and Labrador
PV: Pathogenic Variant
TGC: Traditional Genetic Counselling
